# Review of the Brain’s Behaviour after Injury and Disease for Its Application in an Agent-Based Model (ABM)

**DOI:** 10.3390/biomimetics9060362

**Published:** 2024-06-14

**Authors:** Luis Irastorza-Valera, Edgar Soria-Gómez, José María Benitez, Francisco J. Montáns, Luis Saucedo-Mora

**Affiliations:** 1 E.T.S. de Ingeniería Aeronáutica y del Espacio, Universidad Politécnica de Madrid, Pza. Cardenal Cisneros 3, 28040 Madrid, Spain; luis.irastorza.valera@alumnos.upm.es (L.I.-V.); josemaria.benitez@upm.es (J.M.B.); fco.montans@upm.es (F.J.M.); 2 PIMM Laboratory, ENSAM–Arts et Métiers ParisTech, 151 Bd de l’Hôpital, 75013 Paris, France; 3 Achúcarro Basque Center for Neuroscience, Barrio Sarriena, s/n, 48940 Leioa, Spain; edgar.soria@achucarro.org; 4 Ikerbasque, Basque Foundation for Science, Plaza Euskadi, 5, 48009 Bilbao, Spain; 5 Department of Neurosciences, University of the Basque Country UPV/EHU, Barrio Sarriena, s/n, 48940 Leioa, Spain; 6 Department of Mechanical and Aerospace Engineering, Herbert Wertheim College of Engineering, University of Florida, Gainesville, FL 32611, USA; 7 Department of Materials, University of Oxford, Parks Road, Oxford OX1 3PJ, UK; 8 Department of Nuclear Science and Engineering, Massachusetts Institute of Technology (MIT), 77 Massachusetts Ave, Cambridge, MA 02139, USA

**Keywords:** connectome, brain injury, neurodegenerative diseases, Parkinson’s, Alzheimer’s, dyslexia

## Abstract

The brain is the most complex organ in the human body and, as such, its study entails great challenges (methodological, theoretical, etc.). Nonetheless, there is a remarkable amount of studies about the consequences of pathological conditions on its development and functioning. This bibliographic review aims to cover mostly findings related to changes in the physical distribution of neurons and their connections—the connectome—both structural and functional, as well as their modelling approaches. It does not intend to offer an extensive description of all conditions affecting the brain; rather, it presents the most common ones. Thus, here, we highlight the need for accurate brain modelling that can subsequently be used to understand brain function and be applied to diagnose, track, and simulate treatments for the most prevalent pathologies affecting the brain.

## 1. Introduction

*The goal of this section is to broadly unfold the topic of brain health and its importance while superficially pondering some of the intrinsic shortcomings when attempting to model its behaviour and evolution under certain pathologies.*

The brain acts as an animal’s central computer: it generates knowledge by gathering information obtained through the senses (as a result of external and internal stimuli) and associative processes allowing it to change the environment. Furthermore, it regulates biological constants essential for life, like breathing, body temperature, and heartbeat, and more complex processes such as emotions and conscience. It is the most energy-consuming organ in relation to its size (a fifth of the total in a resting state), despite undergoing evolutionary optimisation in different animal species [1].

According to the World Health Organisation (WHO), two out of ten principal causes of death globally in 2019 were directly related to brain malfunction: strokes (also called cerebrovascular accidents, CVA) and dementia (including Huntington’s chorea; Parkinson’s, Alzheimer’s, and Lewy’s bodies; and many others) [2]. Strokes are caused either by a lack (ischemic, 85% of total [3]) or excess of blood flow (haemorrhagic) in the brain and are thus closely related to the leading cause of death, coronary artery disease (CAD). Dementia is an umbrella term for neurodegenerative illnesses, resulting in the loss of mental capabilities, especially those affecting speech and memory. They can be age-related (Alzheimer’s, Parkinson’s) or not (e.g., Huntington’s chorea), and they are not to be mistaken for neurodevelopmental disorders, which affect the brain in its first stages of growth.

Cancer is another prominent source of mortality worldwide. In fact, brain tumours, despite not being the most common ones (around one-in-fifty diagnoses [4]), are amongst the most fatal (one in three have a five-year survival rate on average in the US, around 5% for the most aggressive types, such as glioblastoma [5]) and are alarmingly prevalent in children under 14 (about one-sixth of total cases as of 2019, second only to leukaemia [6]). Neurological illnesses (including injuries induced by tumours) are the leading cause of crippling disability, and their incidence is increasing, especially affecting ever-ageing populations in the developed world [7]. In psychiatry, the freshly emerged field of pathoconnectomics explores the links between brain wiring degeneration [8] and disorders like depression [9], schizophrenia, or autism [10] or the association between cerebral small vessel disease (CSVD) [11] and cognitive impairment leading to Alzheimer’s [12].

Neurological disorders are very costly not only at a personal level (suffering by the patient and their relatives) but also on an economical one, dealing in billions of euros per year in developed countries where diagnoses are more prevalent [13,14,15,16]. Poor health systems in developing countries make diagnoses and treatment more challenging [7,17,18]—which poses an inequality issue. Although data are relatively abundant, data might not be representative enough [19,20,21]. In order to obtain a better panorama of the current situation, new data managing approaches have become widespread, such as data mining [22,23,24] and machine learning [25,26]—especially graph neural networks [27,28,29,30,31,32,33].

Overall, it is self-evident that the study and characterisation of the brain is a dire need not only for scientific purposes but also, and most importantly, for health reasons, such as the study and treatment of related pathologies for a better diagnosis and hopefully a more effective treatment. Computational neuroscience, i.e., modelling the brain’s function via computers, could bridge the gap between the plethora of unconnected partial data available on the brain by providing a full comprehensible in silico mock-up (digital twin). However, that goal requires multiple challenges to be overcome [34].

Some of these problems come from “traditional” experimental neuroscience (availability of representative data, multidisciplinarity, multiscalar aspects, general complexity, accurate diagnosis), while others have become apparent when trying to manage the obtained data (computational costs, compiling and memory, organisation and classification, limiting mathematical approaches, lack of unified theoretical framework to analyse and compare, brain–environment interactions). Should these issues be successfully addressed, a powerful simulation tool for simulating healthy and ill brains would be available. Thus, the diagnosis and treatment of any condition would be at the doctor’s disposal, minimising difficult, dangerous, and costly in vivo interventions. Additionally, as neuroscience develops as a scientific field, its findings can be incorporated into computational tools (both hardware and software), which may in turn further ease the problem, since the brain remains the most powerful and efficient computer known to man [35].

### Scope and Methodology

**Figure 1:**
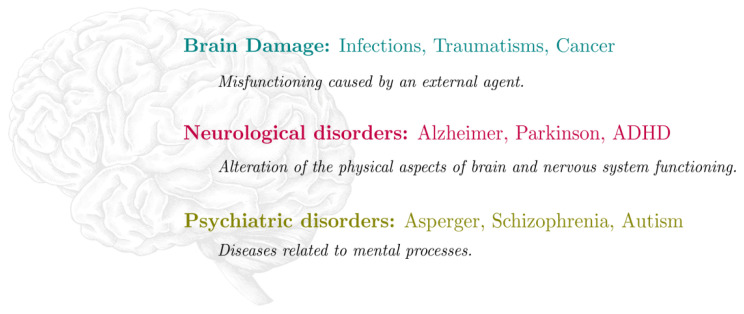
Main aspects studied in the present article.

The purpose of this review article is to briefly showcase the most relevant information gathered about such health issues in relation to the brain’s structural and functional architecture - namely injuries and disorders, see Figure 1 for disclosure. In no way is this article to be construed as a detailed medical handbook; rather, it is a compilation of general brain structure and its illnesses with a detailed enough context to understand the issues they pose from a modelling perspective, with the aim of developing efficient virtual twins for diagnosis and treatment follow-up. As such, some descriptions may suffice from an engineering perspective but still be lacking from a doctor’s.

The employed research methodology is quite straightforward, conducted by searching “[illness/disorder]” AND “(structural/functional) connectivity” in widely known medical databases (e.g., PubMedCentral) and journals (Cell, The Lancet, etc.). In most cases, this ensures that the found publications present the examined disorder from a connectomic perspective, i.e., in relationship with the brain’s structural and functional networks. The authors are aware that this vision does not provide the full picture, but it is enough for the purpose of this article.

After this Introduction underscoring the relevance of brain-related illnesses and trauma in Section 1, a brief note on the brain’s structure and function will be introduced in Section 2. Section 3 will present a general overview of the most prominent causes of brain damage, while Section 4 contains some modelling propositions for the connectome. Finally, Section 5 acts as a summary of this article followed by some research suggestions.

## 2. Mapping the Brain: The Connectome

*This section presents the brain as a collection of its physical (structure) and physiological connections (function), accompanied by some historical context justifying the birth of connectomics as a field of neurosience, which is the lens that every upcoming illness description will be looked at through.*

The first known reference to the brain is the Edwin Smith Surgical Papyrus (Egypt, 1700 BC), although back then, its functions were thought to be carried out by the heart instead, as Aristotle theorised. Although there were some anatomical studies of the brain during Roman and Baghdad Caliphate times, not much else could be conducted until the invention of the microscope in the late 1500s AD. Some nerves (especially related to the sensory systems, such as sight or hearing) were correctly identified, but it was not until the second half of the 19th century that the field work by Broca, Wernicke, and many others began to correctly associate specific areas of the brain with certain tasks (muscles of speech and language comprehension, respectively).

It became apparent that patients who had undergone injury or surgery affecting certain brain zones saw some of their cognitive abilities lessened or entirely missing, so doctors began to fill in the blanks in the brain map. One of the first attempts to fully map brain function was made by Korbinian Brodmann in 1909 [36]. By staining neurons with Nissl’s method, he divided the primate brain into 52 areas (44 present in current human beings and 8 remaining only in related primates) based on cytoarchitectural (cell-level structure) criteria.

Brodmann’s map has been the primary guide for decades, and it is still used on an educational level today, but evidence of its incompleteness is abundant and continuously growing. For instance, it is possible to live—albeit in an altered state and with great difficulty—with a fraction of a brain, as in severe cases of hydrocephalus (partially substituted by water) [37], lobotomies (the extraction of part of it) [38], or even anencephaly (the absence of it altogether) [39]. These cases can be explained through the concept of neuroplasticity: the brain’s ability to rewire its functional and structural connections to overcome injury’s effects. This shows that the spatial distribution of functional areas is more flexible than charts like Brodmann’s would suggest. Neuroplasticity allows for a certain regenerative capacity as well, although humbler in the central nervous system than in the peripheral one (PNS) [40], which often helps patients recover motor coordination after strokes, for example [41].

Significant progress has been made thanks to the dawn of magnetic resonance imaging (MRI) in the 1970s, especially its functional variant (fMRI). Such technology allows us to track brain activity in quasi-real time by measuring changes in blood flow [42], assessing pre-surgery conditions [43], and parcelling brain functional [44] and structural areas [45,46,47]—even on a city [48] or nationwide basis [49,50]. Although relationships between specific tasks and areas in the brain do exist [51] (e.g., memory [52,53]), there are plenty of experimental data suggesting that such a localisationist framework might not be an optimal approach. Many individual actions require the activation of multiple areas in the brain (like language and decision making [54]) and the coordination between them (co-activation [55], newly studied using a plethora of techniques [56]). Therefore, each area is not univocally nor individually responsible for a given action but rather correlated to it.

Seemingly related tasks may be performed by different areas within a brain region; such is the case of facial recognition and evaluation [57]. Activity patterns for the same task differ [58] and even change with age [59] or illness. Although there is a certain consensus on a “default” network configuration in resting states [60,61,62], it is still subjected to individual variations, some of which are associated to pathological conditions [63]. One must also bear in mind that structural and functional pathways in the brain influence each other. On top of that, most brain mappings show a correlation between tasks and activations, rather than the causation of such patterns [64].

Thanks to the experimental work of Santiago Ramón y Cajal in the 1880s, using Camilo Golgi’s methodology, the neuron doctrine gained recognition and could be considered as the beginning of modern neuroscience. It conceives the brain (and the whole CNS by extension) as a network of intertwined but independent neurons, a specific type of cell receiving and transmitting electric signals. Generally speaking, neurons are composed of a soma and an extension covered by myelin (axon) ending in appendices (dendrites) responsible for communication between them (synapse). In mathematical terms, these two main elements could be interpreted as the vertex v and edge e of a given graph G(v,e).

However, the state of the art has long rejected such a primary framework. Current trends in neuroscience focus on the importance of neural circuitry—encompassing the whole nervous system (NS), not just the brain—as opposed to isolated neuronal activity. Also, there are multiple neuron types and morphologies and non-neuronal elements which play an important role in the NS (glia, blood vessels, etc.). Zooming into the brain again, this connectivity between neurons is both structural—physically existing links between neurons—and functional—the links actually used during neural activity—forming the connectome as a whole [65].

Connectomics has shifted the paradigm in psychiatric studies by allowing for the identification of structural and functional measurable alterations in the patient’s brain during and after illness. It relies on quantitative graph parameters such as local network efficiency, clustering coefficients, and global communication path lengths to evaluate damage [66]. MRI has been particularly useful in mapping the cortical surface and structural (diffusion, dMRI) and functional (fMRI) connectivity patterns—even in resting state (rfMRI), although co-activations for the same task and great individual variability cannot be ignored [67].

These two sides, structural and functional, can be mutually affected and/or change due to injury and/or disease or even with mere biological age. Neurons themselves can be classified along these lines: structurally as uni/bi/multipolar/anaxonic, etc., or functionally—within a given brain region—as principal (afferent/sensory, efferent/motor)—projecting to external regions—or interneurons—local wiring. Furthermore, such connections are subjected to neuroplasticity: rewiring (structural [68]) and alternative paths (functional [69]) on a life-long basis [70], especially during growth [71], age [72,73], injury [74,75], and disease [76]. The connectome is also affected by a sort of negative functional plasticity (diaschisis), by which an area distantly connected to another damaged area might see their functions deteriorated by the latter [77] without any apparent linking connections. On top of that, neurons can migrate between brain regions [78] according to their needs, especially—but not exclusively [79]—in early growth stages [80].

Beyond the structural requirements described above, brain cells use chemical signals (neurotransmitters) to communicate. Simplifying this concept, neurotransmitters could be classified as excitatory (e.g., glutamate), inhibitory (e.g., GABA), and modulatory (e.g., dopamine). Such effects depend, at least in part, on the type of receptor where they are acting. Classically, neurotransmission was considered as an anterograde phenomenon, meaning that the neurotransmitter (NT) is released by the presynapse and acts at the postsynapse. Nowadays, we know that such a process is much more complex due to the existence of other types of transmission such as the retrograde modulation of the synapse (e.g., endocannabinoids [81]). Additionally, brain cells respond to other type of messengers, like hormones that allow communication with other organs in the body. In general terms, all these chemical signals could play a key role in plasticity events, and they are susceptible to pathological conditions. For example, Parkinson’s disease is characterised by a deficit in dopaminergic transmission. Likewise, changes in acetylcholine could be associated to Alzheimer’s.

Brain cartography [82] has grown to be popular in recent decades, yielding notable examples [48,83] as a result of a coordinated effort in the sharing and management of huge amounts of data. Such data have been traditionally obtained via physical means, namely haemodynamics [84] (blood oxygen level-dependency, BOLD): computerised tomography (CT), (functional) magnetic resonance imaging (f)(MRI), positron emission tomography (PET), etc. As mentioned, these techniques are costly and subject to instrumental mishandling and/or failure, hence highlighting the need for computational replicas of the brain.

## 3. Brain Damage

*This is the main part of the article, presenting a non-extensive classification of brain-damaging phenomena (traumatic events, illnesses, mental disorders, etc.) that could affect structural and/or functional connectivity in any way, offering mostly qualitative explanations and quantitative when possible.*

Neural cell death takes place regularly in the brain: unneeded neurons are disposed of in a programmed, foreseeable, and controlled way—known as apoptosis [85]. The dying cell implodes, collapsing its cytoskeleton, liberates broken nuclear DNA, and is finally eaten by other cells (phagocytosis). This can be a result of brain development [86,87]—well studied and measured [88,89]—and/or due to structural neuroplasticity (axon retraction, i.e., “pruning” [68]) due to redundancy [90]. Apoptosis can be intrinsic (mitochondrial, BH3-Bax-APAF-1-caspase-9) or extrinsic (death receptor, caspase-8-Bid) [91]. It is a complex process regulated by multiple internal mechanisms involving various molecules such as p75, Bcl-2, and caspase-1 [92] and crucial in fighting cancer—which is, in short, an out-of-control cell multiplication [93,94,95].

Brain damage comes into the picture when the unforeseen deterioration or destruction—necrotic death—of brain cells (neurons) takes place. In this process, the cell’s mitochondria and endoplasmic reticulum swell and break the external membrane. This is due to a plethora of different mechanisms (necroptosis, parthanatos, ferroptosis, pyroptosis, oncosis, lysosomal, autophagic, phagocytic, MitoPore—mitochondrial permeability transition) [91] affecting neighbouring neurons though inflammatory expansion, unlike apoptosis. Some of these processes result in ATP (adenosine triphosphate) depletion—which, in turn, produces the failure of sodium and calcium channels through the neuron membrane. This provokes cell swelling and degradation via proteases and phospholipases, respectively. Be it this way or by mixed lineage kinase domain-like (MLKL: necroptosis), reactive oxygen species (iron: ferroptosis), or inflammation (pyroptosis, lysosomal), the result is abnormal neuron necrosis [91]. As will be explained in the following subsections, these necrotic mechanisms are linked to the advent of various neurodegenerative diseases [96,97] and other neurological disorders such as sleep apnea [98,99].

### 3.1. Acquired Brain Injury

Any brain injury caused after birth qualifies as an acquired brain injury (ABI), thus excluding congenital defects—which will not be covered in this review, due to their case-dependent nature [100,101]. This damage can have an internal (e.g., tumour growth, mental disorder) or external origin (e.g., concussion, accident). The first kind are usually—but not exclusively—caused by injuries and known as traumatic brain injuries (TBI), whereas the latter, non-traumatic brain injuries (NTBI), normally involve a brain illness—not necessarily neurodegenerative. Both have in common the widespread destruction of the cortical areas and white matter tracts and deep brain damage (thalamus, basal ganglia) [102]. Other complications include hydrocephalus, pneumo-encephalus, ventricular enlargement, skull fracture, unconsciousness, sores, bladder infections, pneumonia, and/or multiple organic failure [102].

ABI’s symptoms can be physical (paralysis, headaches, seizures, insomnia, loss of consciousness, aphasia—speech impediment), cognitive (memory loss, impaired information processing, comprehension, or expression), perceptual (disorientation, lack of equilibrium, sight/hearing/touching/smell/taste dysfunction, hyperalgesia—extreme pain sensitivity), or behavioral (irritability, aggressiveness, lethargy/apathy). Neural damage, on the other hand, is more difficult to quantify, as most imaging techniques can only identify and count dead neurons [103] but not neural malfunction. Nonetheless, some tools are available [104]. ABI can be primary—shear/tear of tissue, complete right after the impact—or secondary—more complex chemical, biological, or biomechanical changes hours after the insult, including blood barrier damage, excitotoxicity (glutamate release), mitochondrial dysfunction, or Na+/Ca+ influx [102]. In the most severe scenarios (GCS < 8), it can lead to prolonged coma states or even death.

#### 3.1.1. Traumatic Brain Injury (TBI)

This category includes brain damage caused by physical trauma, i.e., accelerations, shocks, concussions, incisions, etc., caused by a foreign (external) agent, and it is responsible for more than 1 in 3 annual injury-related deaths in Europe. Furthermore, it accounts for 2 million yearly hospitalisations [105], affecting around 70 million people globally [106] (around 1% of the world’s population), typically young males [107]. TBI can be classified into mild (13–15 points), moderate (9–12 points), or severe (3–8 points) via the Glasgow Coma Scale (GCS), composed of three areas (4 verbal points, 5 ocular, and 6 motor). Depending on its causes, it can be further divided into closed (unbroken skull: fast movements, shaking) and penetrating brain injury (open head, e.g., bullets). There is evidence of comorbidity between several mental conditions such as major depression (MD), post-traumatic stress disorder (PTSD), general anxiety, suicidal behaviour [108], and even sleep disorders, back pain, high cholesterol, osteoarthritis, and diabetes [109].

**Closed Head Injury** The most common type of CHI is mild TBI—meaning not immediately life-threatening (around 80% of all diagnoses [105])–a sub-type of closed head injury (CHI) occurring within an intact skull that is caused by bumps to the head or any other action causing fast skull movement (acceleration/deceleration, especially rotational [110]), which may result in physical strain to the brain and/or chemical changes. These accelerations usually involve coup contrecoup: the back-and-forth jarring of the brain against the skull’s inner walls, shearing tissue and resulting in blood vessel rupture, bruising, and swelling.

This kind of injury is relatively common in any physically intensive activity (sports, military practice) or as a result of acts of violence and accidents (traffic, falls). It can cause some of the aforementioned symptoms immediately after injury (peaking within the first 24 h), during which the brain undergoes gliosis—glial cell multiplication, forming scars [110]. Such a process hinders healing but also entails potential long-term effects [111,112,113] such as chronic traumatic encephalopathy (CTE) in the case of recurrent mild TBI, common in sportsmen. In fact, up to 90% of athletes in the US [114] may experience memory and attention impairments, suicidal behaviour, or even cardiovascular complications [115,116].

TBI usually produces three major effects: acute subdural haematoma—associated with traumatic cerebrovascular injury (TCVI) in a limited number of cases (less than 2% [116])—brain contusion, and diffuse axonal injury [117]. Contusions involve brain bruising as a result of coup contrecoup accelerations, mostly affecting the frontal and anterior temporal lobes [118]. These are not to be mixed with concussions, an umbrella term for closed-head, mild TBI involving transient mental effects ranging from confusion to loss of consciousness.

Haematomas are extended contusions causing blood overflow from multiple broken blood vessels (due to brain bruising), common in physical trauma. It can be epidural (EDH) or subdural (SDH). The former involves a rapidly leaking broken artery between the dura mater (third and most external layer in the meninges) and the skull. The latter takes place when a bridging vein ruptures and slowly seeps between the arachnoid (second meningeal layer) and the dura mater. Traumatic subarachnoid haemorrhage (SAH) can also take place if capillaries break and flood the region under that layer. Such a blood volume (arterial or venous) may interfere with the Monro–Kellie principle (the total intercranial volume remains constant) [119,120], resulting in a pressure increase on the rest of intercranial components, namely, brain tissue and cerebrospinal fluid (CSF). That could lead to cerebral oedema (fluid accumulation), the disruption of the blood–brain barrier, and/or diffuse axonal injury, among other undesirable effects.

Diffuse axonal injury (DAI) is the strain/tearing of axons all across the brain due to stresses (compression, tension, shear) occurring during and after TBI in 1–15 mm stretches within a particular distribution [102]. It is provoked by both intense strain (10–50%) and strain rates (10–50 Hz) [117]. This phenomenon is more acute at the junction of grey and white matters with different densities, where the axons are covered in thicker myelin sheaths and surrounded by a drier environment. During stretching, the axon could swell and fracture, increasing its permeability and calcium influx and thus unchaining necrosis [118]. It can also have long-term effects such as greater chances of developing neurodegenerative diseases like Alzheimer’s [121,122,123,124]. DAI is usually detected by MRI, although it takes time, so CT may be preferred for fast haemorrhage identification if the patient needs urgent treatment [125]. Despite CT being less detailed than MRI [102,126], it can be further enriched by gradient echo (GRE) [127] or susceptibility-weighted imaging (SWI) [128]. A major problem when modelling DAI is the fact that it is delocalised all over the brain (hence “diffuse”) and, thus, it is difficult to predict an injury pattern in a deterministic way given a certain traumatic origin.

**Penetrating Brain Injury** Penetrating brain injuries (PBI), although less common than closed head injuries (CHI), are also fairly more lethal. They are caused by external collisions against the skull, which is often fractured, resulting in haematoma and/or intercranial haemorrhage, mostly fatal in the basilar area [107]. Its origins are various (assault, murder/suicide attempts), usually involving physical violence and/or projectiles such as bullets. Consequences can include short-term outcomes like severe trauma in 55% of cases [129] (GCS < 5 for gunshots [130]), generalised haemorrhage, CSF leaking, intracranial infection, aneurysm (50 % lethal [131]), and often death (around 40% [129]—up to 90% if neurological status is poor [131]). Long-term effects include post-traumatic epilepsy and/or seizures for 1 in 2 cases [132].

PBI’s effects vary greatly according to the kinetic energy liberated by the weapon used to inflict the damage $E=mv^{2}/2$, which varies linearly with mass (the heavier the object, the greater the damage) and quadratically with its speed (low if under 300 m/s, medium up to 600 m/s, and high upwards). Thus, light, low-velocity objects like nails or knives are less likely to cause severe damage. Bullets and shrapnel, on the other hand, travel much faster and can increase their already devastating effect—direct by penetration or indirect by shockwaves (causing cavitation)—depending on shape, angle, deformation, or the shredding of the skull and/or projectile inside the cranium.

The rapid evaluation of PBIs is vital to increase the chances of patient survival. Undergoing CT could determine if surgical intervention is viable, be it to heal wounds and/or extract the projectile (except knifes at first [133]), and act ipso facto to prevent worsening scenarios related to hypoxia, anaemia, CSF leaks, hypotension (systolic under 90 mm Hg), or hyperpyrexia (extreme fever over 41.5 °C). Prophylactic anticonvulsants are used to prevent seizures, and special attention is paid to aggravating factors: old age, severe coma (GCS = 3), high intracranial pressure, coagulopathy, and thrombosis [134]. The prognosis depends greatly on the foreign object’s trajectory, being mostly reserved if it crosses the midline, ventricles, or posterior fossa [133] or if it affects the brainstem or both lobes and/or hemispheres [131]. Self-inflicted wounds and pre-hospital intubation and craniotomy/craniectomy are positively correlated with the mortality rate in PBI [129]. Importantly, the laceration of tissue caused by projectile disintegration enhances damage.

As a result of PBI, neural tissue can be either physically deteriorated (sectioned by the foreign object) or dead (insufficient blood flow due to leakage, hypotension, etc.). Damage is usually more localised than in CHI but also more severe, often meaning permanent neurological consequences and death in some cases. Non-trauma-derived infections by the lack of prophylaxis are another common complication. This will be better explained in the next section.

#### 3.1.2. Non-Traumatic Brain Injury (NTBI)

This category includes any brain injury with internal origin, especially infections (meningitis, encephalitis, etc.), poisoning (radiation, lead), lack of oxygen (aneurysm, stroke, heart attack), or any other event increasing internal cranial pressure (e.g., tumours). Sometimes, they can be broadly referred to as ABI, being acute (mainly anoxia/hypoxia-induced: stroke, heart attack, drowning) or chronic (migraines). In general terms, they can potentially spread all across the brain (diffuse injury), targeting the neuron’s structure [102]. Moreover, it can have similar effects to TBI, including coma states [135].

**Infections** Neurological infections, although almost eradicated in Europe and North America, are relatively prevalent in developing countries. The most common way for infections to come in involves the contamination of the cerebrospinal fluid (CSF) in the central nervous system (CNS) via blood (haematogenic), contiguity to infected organs or bones, or neural transmission [136]. Haematogenic contagion caused by pathogenic agents (bacteria, fungi, protozoa, or parasites) in the blood stream is mainly arterial through the junction of white and grey matters (parasitic/bacterial, some viruses). When this happens, there are chances to expand elsewhere, although neurotropic viruses (such as herpes or measles [137]) enter the CNS through the blood–brain barrier (BBB), transcytotic epithelial passage, or leucocyte infestation.

Venous infections are rare, but they can produce notable effects such as schistosomiasis (trematode worms) and (micro)thrombophlebitis (blood clot-induced vein swelling). Transosteal infections originating in adjacent frontal (face, e.g., sinusitis) or temporal areas (such as otitis affecting the petrous bone) can generate intracerebral abscesses or pericerebral collections (extra-/sub-dural empyema). Neural propagation, although unusual, is a vector for viruses (herpes simplex, varicella, rabies) and bacteria (listeria). Lastly, infection can also occur in direct contact with the cranium or vertebrae after a PBI or surgery (such as nosocomial meningitis). However, about 1 in 5 brain infection cases have unknown origin [136].

Most common infections imply inflammation (meningitis—meninges; encephalitis—brain parenchyma) or other alterations in intercranial pressure such as abscesses (intracerebral pus accumulation), which of course implies shear and compressive stresses on axons. Some of them (pyogenic or tubercular abscesses, Aspergillosis, Whipple disease, etc.) can complicate diagnosis by mimicking space-occupying lesions (SOL, i.e., tumours), losing valuable and timely treatment opportunities [138]. In the case of sepsis, damage (inflammation, BBB disruption, hypoperfusion) can have long-term effects (cognitive impairment) [139].

Immunosuppressed patients are particularly vulnerable, having a worse prognosis. Sexually transmitted diseases (STDs) such as HIV or syphilis can have deep neurological impact, most commonly meningitis, but also more severe like progressive multifocal leukoencephalopathy (PML). Some other prominent infections targeting the CNS are those related to helminthic worms, provoking angiostrongyliasis, gnathostomiasis, (neuro)cystercosis, or schistosomiasis, among others [140]. The source of infection could be multimodal. For example, many different parasites can infect human brains, causing eosinophilia (abnormally high concentration of eosinophils in blood [141]). Moreover, infections affecting other organs can reach the brain, like the case of tuberculosis, whose expansion causes 6% of all meningitis [142]. Finally, domestic animals can also be carriers of viral infections causing encephalitis (rabies by dogs, toxoplasmosis by cats).

**Autoimmune diseases** Autoimmune diseases are associated with myelin loss and haemorrhage, the latter coming from within [143] or outside the brain (such as lupus erythematosus [144]), and can cause very similar effects to those of infection (meningitis, encephalitis, epilepsy, vasculitis) [145]. Like infections, they often result in brain damage via anoxia, stress, or chemically-induced necrosis [143].

**Toxic/Metabolic** Toxic and metabolic conditions are intertwined, as they share a pathophysiological description, although a slight nuance can be construed: toxicity can come from internal (metabolic dysfunction) or external agents (toxic substances the patient has been exposed to). Some examples of metabolism-related toxicity targeting the brain are high-pressure oxygen [146], hyperammonemia (excess of ammonia due to urea cycle malfunction or general disorders, especially damaging to developing brains) [147], and several conditions (hepatic failure, hypoglycemia) [148]. In the absence of a clearer nosological classification, both pathogens have fallen under the umbrella term of toxic metabolic encelopathy (TME) [149].

Neurotoxins causing TME can be classified into metallic compounds (e.g., lead, mercury, arsenic) [150], non-metallic inorganic (sulphur, bromine), industrial (e.g., carbon monoxide, aliphatic hydrocarbons [151], cyanide), nerve agents including gases (e.g., sarin), and liquids (e.g., Novichok). These toxins can target the soma (especially heavy metals) and axons (degeneration by industrial chemicals). They produce a separation or loss of myelin sheets, resulting in the abnormal transmission of nerve impulses). Furthermore, they could target specific cell types like astrocytes (abnormal ionisation in membrane activation, processing of toxins).

On the other hand, drugs (e.g., alcohol, cocaine, methamphetamine) and naturally occurring biological agents (mushrooms—*A. Muscaria*, *A. Phalloides*; animals—snakes, spiders, scorpions; microorganisms—botulism [152], tetanus, diphtheria) [153] have a great negative impact on neurotransmission (e.g., serotonine/dopamine depletion) [154]. Drugs can also alter blood flow in the brain. In particular, cocaine and heroin are linked to hypoxia and ischaemia [155]. Thus, the consequences of exposure to neurotoxins vary greatly but often include cerebrovascular accidents (infarct, hypoxia/ischaemia, haemorrhage, vasogenic cerebral oedema), infection, (astro)glial abnormalities, diffuse neural necrosis, and dementia in the long term [149,156,157].

Radiation poisoning, either accidental or planned (e.g., radiotherapy as a treatment for tumours) involves exposure to ionizing agents, and so it can have dreadful effects in brain tissue through four main mechanisms: vascular damage, astrocyte extinction, cytokine alterations and stem cell death (mainly in the hippocampus [158], cerebellum and cortex) [159]. Acute symptoms include oedema, vascular hyalinisation, myelin depletion, inflammation, ischaemia, necrosis, dementia [158,160] and even tumour progression—a common adverse effect in oncological radiotherapy [161,162] that is dose-dependent [163] and not so easy to identify [164].

**Vascular** Although they can occur after trauma (TCVI [116,165]), cerebrovascular injuries can also originate from inside the skull via the defective oxygenation (hypoxia/anoxia) of blood vessels within brain tissue, leading to necrosis: a stroke, the second leading death cause worldwide [2,166]. The incidence of stroke, also known as cerebrovascular accident/insult (CVA/CVI) or apoplexy, is steadily increasing, especially for populations over 75 years old. It can be ischaemic (brain infarction due to insufficient blood supply via vessel occlusion) or haemorrhagic (intracranial blood spilling via vessels or aneurysm rupture).

The former are by far the most common—around 85% of all cases [166,167]—and they are sometimes anticipated by transient ischaemic attacks (TIA) [167], falling into several aetiological categories: large-artery atherosclerosis, cardiogenic embolism, small vessel occlusive disease, and determined or undetermined cause. Haemorrhagic stroke can be further classified into intracerebral (ICH, within brain) and subarachnoid (SAH, between inner and outer meninges), as previously mentioned. Its symptoms include high blood pressure, incoherent speech, and motor impairment. Risk factors can be fixed age, genetics (race, family history), sex, modifiable hypertension (causing haemorrhagic strokes), diabetes, nutrition (diet, obesity), alcohol, sedentary lifestyle, or cardiac conditions, among others [166,167].

The brain’s function is rapidly disturbed during strokes [168], either by tissue loss (necrosis) or abnormal patterns in the interhemispheric connectivity [169]. Paradoxically, these changes play also an important role in recovery [170,171,172] by promoting small-worldness in neural networks, i.e., high nodal clustering (many local hubs—specialisation) and short inter-nodal path lengths (faster neurotransmission efficiency, integration) to alleviate necrosis and functionally rewire the remaining neurons [173]. Studying the brain’s angiome (blood vessel network) can provide meaningful information when facing stroke detection and therapeutics, since its mappings are easier and more developed than those of the connectome [168], which it directly affects.

**Cancer** Tumours, also known as neoplasms, are lumps of abnormally growing cells. They can be benign (non-cancerous, slowly growing, and unlikely to spread) or malignant (cancerous, rapid growth, and prone to propagate). Brain tumours originate mainly in intercranial tissue or the meninges (meningiomas), although they mostly [174] come from different adjacent organs (metastasis); such is the case of melanoma (skin cancer), prostate/breast cancer, lung cancer, or Hodgkin’s lymphoma (lymphatic system cancer). Although rare amongst cancers (2% of total [4]), brain tumours have a great potential to be lethal (around 10% 10-year survival rate [4]) or produce long-term sequelae. Risk factors include ionizing radiation, immunosuppression (infection, allergy), chemicals (N-nitroso compounds are mutagenic and trespass the BBB), and head trauma.

Symptoms are very general (e.g., headache), although the apparition and evolution cognitive impairment (e.g., aphasia) and seizures are usually more telling [175]. The diagnosis is made through gadolinium-contrasted MRI. This tracer is commonly used when the BBB is already broken by a malignant neoplasm. Interestingly, alternatives like fluorodeoxyglucose (FDG) and labeled aminoacids might be able to detect low-grade gliomas in time before they develop into glioblastomas [176].

The existence of more than 100 types of brain tumours makes their classification difficult, having undergone recent restructuring to include molecular biology information, e.g., mutations related to proteins IDH, B-raf, or MGMT, in their traditionally histologic criteria [177]. If they originate within the CNS, they are called primary brain tumours, the most common being gliomas (75% of diagnoses [178]) originating in glial cells or supportive tissue in a circumscribed or diffuse manner: astrocytomas on astrocytes (anaplastic—grade III; glioblastoma multiforme—grade IV), ependymomas (ventricles, spinal cord), oligodendrogliomas (CNS, myelin production), or brain stem gliomas. Some of them can be localised (astrocytomas), while others are diffuse and can easily metastasise (glioblastoma multiforme) [175]. Other brain tumours without glial origin are medulloblastomas (developing nerve cells), meningiomas (meninges), schwannomas (Schwann cells), craniopharyngiomas (pituitary, near hypothalamus), and germ cell (gametes) tumours.

Their effects on the connectome are multiple: mechanical (pressure by tumour growth on the remaining healthy brain tissue plus sometimes oedema, damaging it), chemical (protein mutation which deteriorates synapses), vascular (thromboembolism), and necrosis—with subsequent functional impairment depending on the area. As mentioned above, neurotoxicity is both a common origin and byproduct of some tumour treatments (radiotherapy, chemotherapy), identified as a possible trigger for glioblastoma [179].

### 3.2. Neurological Disorders

Diseases are a common origin for brain damage that is not injury-related but rather a result of a prolonged condition, developing over a mid-/long-term basis. They are presented in a separate group to differentiate them from incidental, acute insult (previous section). They include pathologies like epilepsy and dementia, neurodegenerative illnesses, and psychiatric/motor disorders.

#### 3.2.1. Epilepsy

Epilepsy is a chronic, non-contagious neurological disorder causing recurrent (at least two episodes within 24 h) uncontrollable, unprovoked seizures, thus excluding those induced by traumatic brain injury, electrolyte disequilibrium, or concomitant diseases. It affects 1% of the world population [180], and its onset can be focal, generalised—affecting both hemispheres, motor or non-motor—or unknown. Aetiologically, it can be genetic (mutation affects the management of voltage-dependent sodium channels [181]), structural (network abnormalities), infectious, metabolic (biochemical changes), immune (induced inflammation), or unknown. Although it is rarely lethal, epilepsy can have long-lasting neurological effects [182].

Its incidence depends on age (prominent during childhood—75% of diagnoses [183]—and adolescence), gender (more common in males), the availability of medical devices (compromising in developing countries [180]), socioeconomic status (the lower the income, the higher the chances) [184], and developmental conditions (autism is correlated). Its symptoms vary greatly with the originating cortex area: visual phenomena such as blinking, vision loss, and hallucinations (occipital lobe); clonic/tonic motor responses (precentral gyrus, frontal lobe); sensory, e.g., numbness (postcentral gyrus), etc. [183]. They may be preceded by a warning or omen, known as “aura”.

Epileptogenesis involves an imbalance between excitative and inhibitive neuronal pathways as a result of uneven activation potentials creating a synchronised wave of excessive neuron firings [180,181]. The malfunction of enzymes (ATPase) and/or glia regulating the extracellular ion concentration (e.g., potassium overload) may produce neuron depolarisation and action potential discharge, leading to seizures, which raise potassium levels even further.

This creates a cascade effect (epilepsy), which also strengthens dendritic echoes, enabling further activation [185]. GABAergic synaptic transmission may lead to depolarisation if its controlling ions (e.g., chloride) are altered. Aberrant neural network synchronisation requires not only the aforementioned excessive discharge but also triggering events such as paroxysmal depolarisation shifts (PDS) of cortical pyramidal cells or neuroplasticity itself—for instance, axon collateral sprouting [181]. Although anti-epileptic drugs are effective in 2 out of 3 cases [186], insight into the connectivity implications of epilepsy [187] is greatly needed for more effective treatment strategies. Synchronisation may be limited to the lamellar axis, according to some studies [188].

#### 3.2.2. Neurodevelopmental Disorders and Disabilities

This subsection covers diverse non-degenerative neurological disorders with early onset (childhood, even premature) impairing intellectual tasks on a daily basis. They affect a small but appreciable percentage of the world population (1–5%), usually more males than females (ratio of 2:1 to 4:1) [189,190,191].

A subject with the (Gilles de la) **Tourette syndrome (GTS)** undergoes diverse motor and phonic tics—involuntary and uncontrollable by definition. It usually appears by the age of 10 in 0.5–1% of children, and its symptoms lessen over time. Remarkably, the patient’s quality of life can be greatly compromised when combined with social isolation or concomitant neuropsychiatric disorders (ADHD, OCD, anxiety, or depression) [191]. GTS has a multifactorial origin, where genetics play an important role (around 77%)—involving the SLITRK1 family of proteins—although environmental and immune factors work together with neurochemical changes (dopamine, GABA, glutamate, serotonin).

GTS alters a wide range of brain circuits, including emotion-related limbic structures (hippocampus, amygdala, and prefrontal cortex to ventral striatum) and regions involved in goal-directed behaviours (ventral medial prefrontal cortex to caudate nucleus) [191]. More precisely, it concerns structural connectivity alterations (basal ganglia) and functional abnormalities (frontal and cingulate regions) [192]. However, having these perturbations as markers for diagnosis [193] can prove deceiving, as symptoms and networks evolve significantly with age. Treatment usually implies behavioural therapy combined with pharmacology only if tics produce pain or injury [191]. The alleviation of symptoms—namely, tics—is positively correlated with certain connectivity patterns in limbic or associative networks, thalamus, caudate, and cerebellum, for instance [194].

**Attention-deficit/hyperactivity disorder (ADHD)**—once known as hyperkinetic disorder (HKD) [190,195]—is a cognitive disorder characterised by the developmental impairment of executive functions (EFs) due to a pervasive lack of attention, hyperactivity, and impulsivity. In particular, ADHD subjects suffer from a deteriorated working memory, inhibitory control, reward processing, and/or planning. It affects around 5% of children [190,196], with a typical onset before 7 years of age and perhaps from birth [197], and 2.5% of adults [196] worldwide. Symptoms persist after puberty in up to two-thirds of diagnosed children [197,198], although they tend to lessen over time [196].

Despite not being considered a learning disability in and of itself, it can coincide with dyslexia or dysgraphia and neuropsychiatric disorders (mainly behavioural [197], but also anxiety, depression, bipolar/personality disorder [199], etc.) which can greatly compromise an accurate diagnosis and an effective treatment. Its aetiology is manifold, involving genetics (twin comorbidity, premature birth [197]), environmental factors (exposition to lead, early adversity [200]), and physiology (brain connectomics). Cognitive, motor, and affective impairments in ADHD have a measurable [201,202] origin in both structural (overall 4–5% less white matter and reduced grey matter cortical thickness [202]) and functional connectivity (decreased between the dorsal anterior cingular cortex and the default mode network [203], with spurious activity in the DMN [202]). Functionality can be improved via medication [201], although its effects are difficult to quantify due to individual differences and reproducibility issues [204]. Pharmacological treatment involving dopamine agonist stimulants—e.g., methylphenidate and atomoxetine—have a 60% success rate in adults [198]. Engagement in psychotherapy (behavioural, counselling) improves the prognosis in the long term.

**Learning disabilities (LD)** encompass several deviations in standard neural development affecting 1 in 20 [189] children and adolescents in regards to areas such as language expression (verbal and non-verbal), reading (dyslexia), writing (dysgraphia), mathematics (dyscalculia), or movement coordination (dyspraxia). By definition, they are not provoked by mental retardation, emotional disturbance, or cultural differences, although they may present comorbidity with other disorders such as ADHD and social impairment [205]. Its aetiology has not been fully deciphered, but it involves a genetic component (X-chromosome syndromes). LDs affect the brain structurally—the abnormal symmetry of planum temporale [189]—and functionally—the right hemisphere and parietal and occipital areas are more active than the left hemisphere and frontal regions [206,207,208], confirmed by DTI and fMRI [209].

**Dyslexia** is arguably the most common learning disability and thus the most studied. Regarding the connectome’s structure, it has been associated with greater anisotropy in white matter (at a thalamic level) that is scalable by age [210] and lower modularity in reading and resting-state networks and in between them [211]. Aberrant functional brain connectivity (e.g., in visual areas [207,212]) is characteristic of dyslexia, worsening reading performance. Neurostimulation has been suggested as a treatment strategy [208]. In general terms, LDs result from functional abnormalities rather than structural ones; such is the case for coordination disorders [213] and mathematical [214], spatial, and nonverbal impairment [215].

**Intellectual disability (ID)** entails a significantly deficient intelligence, i.e., intellectual quotient (IQ) under 70 (100 being the average), which manifests as daily life difficulties in communication, logical processing, socialisation, and self-care. It is present in 1 to 3% of the world’s population, with 85% of cases being mild (50–70 IQ) [216]. Early developmental delays in language and/or motor functions are relatively prominent among children and they do not imply disability per se, unless they grow stronger over time. Multiple apparently unrelated genetic syndromes (e.g., Klinefelter’s, Fragile X, Prader–Willi, or Down syndromes) are associated with ID, so a differential diagnosis requires further testing. Those conditions comprise a range of concomitant symptoms with no defined treatment nor univocal aetiology, albeit with deep biological causes in the most severe incidences [216].

**Down syndrome (DS)** is perhaps the most known type of intellectual disability [217]. It frequently implies cardiovascular disease—congenital in half of all cases and the leading cause of patient mortality, worsened by related pulmonary, endocrine, and metabolic diseases [218]. Adding to that list are issues like dementia (up to 80% by age 65), sleep disorders (65%), dysphagia (55%), visual issues (57%), hypothyroidism (50%), leukaemia [219], and many others [220]. The study of DS not only improves the quality of life for patients but also sheds light on the genetic component of associated illnesses such Alzheimer’s [219,221] or congenital heart disease [222]. Its origin lies in genetics: a third abnormal copy—partial or complete—of chromosome 21 (pair 21 trisomy) is present in the patient’s genome [220], mostly explaining both its aforementioned side effects and variability in the clinical picture.

Multiple studies have tried to link the symptoms and evolution of DS to alterations in brain network configuration. An appreciable brain matter loss is found in post-mortem examinations, and structural changes are detectable even before birth (e.g., excessively large ventricles) [217] or via MRI (changes in white matter like the corpus callosum) [221]. Functional connectivity is diminished and less efficient than healthy controls in adulthood [217] but seemingly higher during youth—although this is disparate [223], which means that long reaction time and low accuracy are observed. In graph theory terms, functional brain networks in DS patients show a shorter average path length and increased global efficiency. However, disrupted connectivity in the supplementary motor cortex, frontopolar, and pre-motor areas pose a great disadvantage in contrast [224].

#### 3.2.3. Neurodegenerative Diseases

Neurodegenerative diseases are becoming increasingly common worldwide, especially in developed countries where life expectancy is higher and thus dementia is more likely to appear [225]. They imply a progressive and inevitable deterioration in the nervous system (both central and peripheral [226]). Such damage causes neurological dysfunction, ranging from memory loss, e.g., Alzheimer’s, to motor impairment, e.g., sclerosis and Parkinson’s. They can be classified according to aetiology into amyloidoses (Alzheimer’s, Creutzfeldt–Jakob’s), tauopathies (Pick’s disease, CTE), alpha-synucleinopathies (e.g., Lewy bodies), and TDP-43 proteinopathies (e.g., sclerosis) [227]. Although very diverse in nature, they all share some common features. First, instead of a static neuronal loss—typical for metabolic/toxic NTBI—necrosis progressively affects certain cells due to their vulnerability and spreads according to the brain’s neural pathways [227]. Second, they are related to a chronic immune malfunction causing general inflammation [228].

**Dementia** Dementia is the gradual loss of mental capabilities such as thinking and judgement. It affects around fifty million people globally—mainly over 65 years old [229]—presenting behavioural changes and/or deficits in communication, orientation, and memory. While cell deterioration is part of the normal ageing process, dementia implies abnormally high neural necrosis. The only attainable treatment consists of the slowing of decay, which will ultimately make the patient dependent on others to varying degrees. Some risk factors are depression, sedentary lifestyle, diabetes, TBI, or alcohol [230]. The most common dementia-associated illnesses are Alzheimer’s and Lewy’s bodies, although their origins can also be vascular (strokes), STD-related, or traumatic (CTE). Dementia is not to be mistaken for delirium, which is sudden, transitory, and mainly distorts attention mechanisms.

**Alzheimer’s Disease (AD)** is the most prominent type of dementia (half of all cases [231]), affecting around 5% of the European population [232]—a significant fraction, due to their advanced life expectancy. Amyloid-β plaques and neurofibrillary tangles (NFTs) are generated by the accumulation of amyloid β-peptides and hyperphosphorylated τ-proteins (and/or demyelinisation [233,234]), causing inflammation and the degeneration of brain tissue. This becomes apparent via excessive necrosis and brain volume changes. For example, the gyri shrink, while the sulci grow, with an overall volume loss of up to 50% [97]. AD can also be the result of mutation (genes APP, PS1, PS2), though very rarely (less than 1 in 20 cases).

AD patients showcase deterioration in memory and thinking processes, together with emotional lability. This is likely produced by diffuse neuronal loss, synaptic degeneration (correlated with NFT distribution), and reactive gliosis (abnormal astrocyte growth) [97], altering the complex cellular micro-environment in the brain [235]. It starts as a localised neural loss in brain areas such as the locus ceruleus but eventually becomes diffuse, reaching other regions like the amygdala, the hippocampus, or the frontal cortex. As cells die all over the brain, neural pathways between tend to stretch, while local clustering remains in the unaffected areas, yielding an efficiency loss as “small-world” characteristics fade away due to sparsity [236] and selective hub vulnerability [237]. Nevertheless, such values depend on user-defined constraints (sample size, brain area, measurements) [238].

The disease’s progress can be tracked via synaptic activity, namely the post-synaptic density protein PSD-95 [239,240,241] and other biomarkers [242]. Enhancing neuroplasticity [76], using some immunotherapeutic drugs [242], and boosting the endogenous cannabinoid system (ECBS) in the brain [81,243] can help slow down the advancement of AD, especially in its prodromal (i.e., early) stages. A precocious diagnosis [242] and realistic computational approaches [237] can prove important in treatment.

**Dementia with Lewy bodies (DLB)** is the result of the accumulation of α-synuclein protein in the brain, creating deposits (Lewy bodies) which disturb the brain’s chemical balance. Although not as common as AD, it represents a sizeable share of cases (around 5–7.5% of all dementias [244]). It is one of the two diseases caused by Lewy bodies, the other being Parkinson’s (PD), discussed in the next section.

**Movement disorders** Under a relatively new and phenomenological medical category [245], this section includes the most common neurodegenerative diseases affecting motor abilities (e.g., walking, standing) as a result of a disruption or dysfunction to coordination between the CNS and muscles. According to the movement’s disruptive expression, they can be further subdivided into hyperkinesias (excessive), dyskinesias (unnatural)—jerky or not— hypokinesias (decreased reach), bradykinesias (slowness), akinesias (absence), and abnormal involuntary movements [245]. Although this subsection focuses on prolonged disorders prominent in advanced ages (over 65 years), transitory movement disorders, such as tremors, dystonia, or tics, are not uncommon in younger patients and can result in early Parkinson’s misdiagnosis [246].

**Parkinson’s Disease (PD)** produces hypokinesia and bradykinesia, among other non-motor symptoms, stemming from Lewy’s bodies (α-synuclein deposits), presenting high comorbidity with LBD. Importantly, such confusion greatly hampers an accurate (and timely) diagnosis [244], which already requires very high-fidelity MRI [247]. These deposits provoke the localised necrosis of dopaminergic neurons in the substantia nigra pars compacta, with the consequential dopamine underflow to the striatum [248]. About 10–15% of all cases are genetic, and they can originate from prion diseases and perhaps metabolic iron accumulation [249] as well.

PD could be considered as an axonopathy characterised by synaptic dysfunction and reduced structural (e.g., white matter) [250,251] and functional connectivity, particularly in the basal ganglia [252]. Some studies suggest that this impaired connectivity is present in prodromal symptoms of PD, like rapid eye movement sleep behaviour disorder (iRBD) [251]. Variability among patients undergoing different stages, treatments, and severities of disease can prove problematic when comparing to healthy individuals [253], despite some praiseworthy attempts [254]. Treatment usually involves L-DOPA (levodopa or l-3,4-dihydroxyphenylalanine), which crosses the BBB to increase dopamine concentrations in the brain.

Lastly, one must not mistake PD for **ataxia**, which is an acute lack of coordination in different muscles affecting gait, speech, and eye movement. These effects come as a result of nervous damage, mainly cerebellar and reversible in some cases [255]. Spinocerebellar ataxias (SCA) are a group of mostly genetic [256], autosomal dominantly inherited neurogenerative disorders, encompassing tens of types with different prognoses and treatments [257].

**Huntington’s Disease (HD)** is a rare, genetic (autosomal dominant) disorder affecting the CNS—especially striatal areas—and showing symptoms like chorea (involuntary, fast, and abrupt muscle movements) and psychiatric degeneration, including dementia. It can manifest itself at any point in the patient’s life, with no noticeable clinical indications until then [258], although most diagnoses happen between 30 and 50 years of age, inevitably leaving to full dependency and death (most commonly by pneumonia or suicide) [259]. HD is caused by a cytosine–adenine–guanine (CAG) trinucleotide repeat (more than 36 times) in the huntingtin (HTT) gene on chromosome 4p, frequently treated with dopamine receptor blockers [259].

In terms of structural connectomics, HD provokes impaired capacity for inter-nodal information processing, characterised by a decrease in nodal betweenness centrality (i.e., reduced relative importance of certain nodes within the network) and the clustering coefficient [260]. This can spread throughout the brain as the mutant protein propagates, which explains white and grey matter deterioration—predictable via graph theory [261]. HD also disrupts functional connectivity in subcortical and default mode networks (brain regions active during passive, non-externally stimulated tasks, e.g., remembering). In some brain areas (e.g., putamen), functional connections are further impeded as the CAG repeat length increases, whereas the contrary is true for other regions (calcarine to middle frontal gyri) [262].

**Prion diseases (PrD)**, also known as transmissible spongiform encephalopathies (TSE) are rare neurodegenerative, deadly illnesses caused by misfolded proteins (prions) $PrP^{C}$ mutating irreversibly into $PrP^{Sc}$. Such misfolding causes neuronal necrosis, vacuolation, and the abnormal activation of microglia and astrocytes [263]. This can take place in humans and other animals (including cattle like sheep and cows), spreading through all organs but especially prominent in the CNS. Their incubation process is long (up to decades), during which those proteins accumulate and create microscopic holes in the brain, transforming its tissue into a sponge (hence, the name).

PrD can be sporadic, spontaneous, and unpredictable (e.g., Creutzfeld–Jakob disease (CJD)), and it can be familial (genetically transmitted, e.g., fatal insomnia) or acquired (through the introduction of contaminated tissue into the patient, e.g., kuru). CJD is the most common prion disease, affecting one in a million yearly (85% sporadic, 10–15% familial [264]), manifesting in young adulthood if acquired (vCJD) and senescence if sporadic (sCJD) [265]. Although uncommon and heterogeneous in aetiology and diagnosis, the study of prion diseases can potentially shed light on the role of protein misfolding in more widespread neurodegenerative diseases such as Parkinson’s and Alzheimer’s [266]. Moreover, its transmission along connected structural pathways can be modelled as graph diffusion [267].

**Multiple sclerosis (MS)** can be considered a neurodegenerative disease [268,269] in its latest stages, after initial autoimmune inflammation in the CNS [228,270,271]. “Sclerosis” means “abnormal hardening of body tissue”. Its ultimate aetiology is unclear. It involves multiple genes increasing susceptibility along with some environmental (ultraviolet B exposure), pathological (Epstein–Barr virus) [271], and genetic factors (highest incidence among European and North American populations [269,270]). It is usually diagnosed in early adulthood via MRI revealing several white matter scars/plaques and chronic CNS inflammation. MS can be intermittent (relapsing–remitting, 85% of cases) or chronic, with chances of drug-induced remission (secondary progressive MS) or not (primary progressive or progressive relapsing MS) [270].

MS affects mainly optic nerves, the brainstem, and the spinal cord [271], provoking demyelination and neuronal loss through axon deterioration [270]. Although remission can happen within hours or days, it is never complete because the neuronal reserve is progressively depleted—hence the neurodegenerative nature of the illness, despite partial remyelination [271]. Primary progressive MS entails ataxia and progressive cognitive and visual failure [271]. Lesions appear as the illness advances, affecting the connectome’s structure and function through the abnormal activation of frontal regions or hippocampus for memory tasks and upsetting the default mode network (DMN) in resting states. However, its direct links to cognitive impairment have proven difficult to clarify [272,273].

Functional connectivity remains a benchmark for studies about MS nonetheless, with special attention to network efficiency indicators [274,275], e.g., on working memory, subjected to patient heterogeneity [276]. Most observations have something in common: altered connectivity in deep grey matter areas, lower brain modularity, hemispheric skewness, and task-independency [277]. Treatment strategies have rapidly improved in a palliative sense but continuously fail to remedy continuous neurodegeneration [278].

**Amyotrophic lateral sclerosis (ALS)**, also known as Lou Gehrig’s disease, is a rare (1 in 100,000 [279]) neurodegenerative disease targeting mainly motor neurons in the brain (upper, in the frontal lobe), the spinal cord (lower), and the brainstem. It is incurable, and patients usually perish 2 to 5 years after diagnosis (usually after 60 years of age [279]) due to the malfunction of the diaphragm (breath) and/or swallowing muscles (nutrition). Although it starts focally, ALS often spreads to other body parts. It aetiology can be autosomal inheritance (10%) by a hexanucleotide repeat expansion of gene C9orf72 (between one-third and one-half of familial cases, although there are at least other 25 genes involved [280]) or sporadic (unclear). Half of all patients experience extra-motor conditions like behavioural changes (apathy, irritation), language impairment, or executive dysfunction, and 1 in 10 show signs of frontotemporal dementia [281].

As ALS unfolds, the assortative networks (between similar nodes) typical for a healthy connectome [282] are dismantled, resulting in a loss of network efficiency. This translates into a functional impairment, predictably with more intensity within the motor cortex [283]. Overall, patients show a decreased functional connectivity in the cortex (right orbitofrontal, left interior frontal) and the corpus callosum [284], as well as an enhancement in the right angular, parietal cortex and the frontoparietal networks [285]. All these changes could be interpreted as compensatory mechanisms—surrogates for lost or damaged brain regions.

### 3.3. Psychiatric Disorders

This category includes non-neurodegenerative disorders affecting mental health, commonly referred as mental disorders, usually treated by psychiatrists. They can be defined as clinically significant disturbances in an individual’s cognition, behaviour, or emotional processes affecting their normal mental function and listed in the Diagnostic and Statistical Manual of Mental Disorders (DSM). They are projected to be one of the biggest health concerns of this century, and they usually develop from infancy and early adulthood (by mid-20s) [286], with diagnoses peaking around 15 years of age (adolescence) [287].

An early treatment could very much determine the future evolution of the illness and the patient’s life quality in subsequent years. Whereas neurodevelopmental disorders (mental disability, e.g., Down syndrome) and anxiety are diagnosed in early infancy (around 5 years), obsessive–compulsive and eating disorders bloom during adolescence (around 15 years). They can bring long-lasting by-products like addiction/substance abuse emerging in the early 20s [287]. Unlike neurological disorders such as the ones listed in the previous section, these have been traditionally diagnosed solely through behavioural changes, although they can be tracked by perturbations in structural and functional connectivity in the brain have been associated with schizophrenia, major depression, bipolar disorder, and autism, among others [288].

**Schizophrenia** is a chronic mental disorder affecting 1% of the population and growing in incidence in developed countries [289], whose characteristic symptoms include hallucinations, paranoia, and social handicap [290]. It may occasionally recede (via therapy and drugs) or become incapacitating. This illness involves a neurochemical imbalance by which the normal course of neurotransmitters (e.g., dopamine) is altered, mainly along four pathways: nigrostriatal (from substantia nigra to striatum), mesolimbic (ventral tegmental area to limbic structures), mesocortical (ventral tegmental area to cortex), and tuberoinfundibular (hypothalamus to pituitary gland) [291].

Its aetiology is diverse, from obstetric problems to genetic factors and environmental triggers (social isolation, trauma). During the illness, the connectome undergoes important changes, including left frontal lobe hyperactivity and [292] network randomisation as clustering decays due to neural path over-shortening [293]. Moreover, there is a distortion of the characteristic “small-world” properties (numerous and balanced local clusters) in healthy brains [294] and altered hierarchies in functional connectivity [295].

**Major depressive disorder (MDD)** is a persistent (at least 2 weeks) and debilitating sensation of sadness or melancholy associated with anhedonia, lack of sleep, and cognitive impairment [296]. It affects 1 in 6 people across their lifetimes [297]. It is the second most devastating burden in terms of disability-adjusted life years (DALY). This includes years lived with disability (DLY) and years of life lost to premature mortality (YLL), mainly due to suicide (50% of them are depression-induced). MDD presents comorbidity with diabetes, heart diseases, and strokes. It implies a large genetic component (about 35% [296,297])—although it is not single-handedly attributed to any specific gene—which often comes with an array of depression-reinforcing behavioural traits (conflict avoidance, pessimism, anxiousness) [296].

In MDD, there is a decrease in functional connectivity among fronto-amygdalar [298], somatomotor [299,300], executive control, and default mode networks in resting state [301]. The latter has been linked to social dysfunction [302], being potentially recoverable by electro-convulsive therapy (ECT) [303] and medication [304] and used as a relapse predictor [305]. That being said, such alterations have a potential for reversal [306].

**Bipolar disorder (BD)** combines depressive and manic episodes characterised by sensations of grandiosity, good mood, and overconfidence [307], on top of chronic mood swing cycles that may become crippling. According to the severity of these manic events, BD can be type I (acute, severe, followed by delusions and hallucinations in 75% of cases), type II (less severe, hypomania), or a cyclothimic disorder (recurring depressive and hypomanic states for over 2 years) [308]. It affects 1 in 100 people worldwide and lists among the leading causes of disability for young patients, yielding cognitive and behavioural impairment which may induce cardiopathies [309] and/or lead to suicide during the worst episodes [310]. Connectivity issues are similar to those of MDD, adding an important decoupling between functional and structural pathways that is strongly correlated with suicide attempts [311,312].

**Autism spectrum disorder (ASD)**, formerly known as pervasive developmental disorder (PDD) [313,314] or “childhood schizophrenia”, [315] involves a combination of social communication issues and recurring behaviours, commonly with an early onset (around 3 years of age) and followed by sensory anomalies and sometimes intellectual disability. It concerns about 1 in 100 people [316] worldwide, although its prevalence is higher in wealthier countries [314,317]. It is mainly hereditary, with a plethora of associated genes and proteins [318], some of them locally inhibiting neural connectivity [319,320], and its presents high comorbidity with other neurological (ADHD, epilepsy) and psychiatric disorders (depression, anxiety) [314].

In contrast to most disorders disclosed in this article, it has been found—through fcMRI, MEG, and EEG—that ASD showcases an excessive functional connectivity (hyperconnectivity) on a network and state basis. This depends on age, although some life-long effects exist, e.g., parietal and frontal hyperactivity (linked to repetitive behaviour in early development) and long-distance hypoactivity [321], depending on the analysed frequency [319]. Nonetheless, this hyperactivity is not necessarily reflected on a structural basis; rather, the inverse is true: brain overgrowth during development gives adults with autism a delayed long-distance connectivity (less efficient). Despite the general tendencies, individual variability [322] and symptom severity [323] play a crucial role in connectivity measurements. In spite of the proof of increased network efficiency markers such as betweenness centrality [324,325], there is contradictory evidence in regards to ASD-induced frontal hyperactivity [326].

**Asperger’s syndrome (AS)** is a chronic neurodevelopmental disorder related to the autism spectrum [327], presenting similar symptoms and connectivity disturbances to those of broad “autism”, except for a greater intelligence on average and general absence of dysphasia (impaired propositional language) [328]. On the contrary, patients tend to develop quite a structured form of language—albeit delivered in an uncommon fashion [327]. Differential diagnosis from social phobia and schizoid personality can prove difficult [329]. There is no apparent structural difference between control brains and AS patients but, functionally speaking, they have higher global efficiency (greater transfer speed) and lower network segregation (transitivity) and resilience (assortativity) [330].

**Obsessive–compulsive disorder (OCD)** is a mental health condition described by repetitive, ritualistic behaviours (checking, washing, counting) to such an extent that patients feel anxiety and fear if they do not undertake them. Intrusive and unpleasant thoughts are characteristic as well. It may be genetically inherited, appearing in early adulthood (22–36 years), and is often misdiagnosed [331]. It is not fully curable, but existing treatments include serotonin re-uptake inhibitors and cognitive/behavioural therapy or even surgery [332]. Treatment can ease symptoms, greatly improving daily life functionality [331]. The incidence of OCD is high among close relatives like twins, especially monozygotic twins (up to 87%) [331]. It is frequently associated with other mood or movement disorders and cortical lesions, even as a result of pregnancy or infection (streptococci) [332]. Its severity can be measured via the Yale–Brown scale.

Structurally speaking, the aforementioned cortical lesions may result in a decreased brain volume, greater grey matter density, or abnormally high-degree connectivity (local in orbitofrontal cortex and putamen, distant in the subthalamic nucleus) [333]. Functional activity is enhanced in corticostriatal networks [334] but impaired in the temporal lobe [332,334], lateral prefrontal cortex, and ventral striatum [334]. Additionally, functional disconnectivity patterns have been observed between some brain regions (striatum–cortex, striatum–thalamus, fronto-limbic-anterior cingulate cortex) [335].

**Anxiety disorders** are the most common psychiatric subtype, involving a persistent sensation of fear and/or worrying in the absence of a clear triggering risk or danger, affecting around 1 in 15 people globally [336]. Anxiety can be treated through a combination of pharmacological (selective serotonin and serotonin–norepinephrine reuptake inhibitors, benzodiazepine [337]) and psychological therapy. There are multiple diagnosis scales available [338] for the evaluation of its severity, with Hamilton’s being the most widely used. It has been linked to structural alterations in the cingulo-opercular network and their connections with the ventral, visual, and default networks in children [339]. Functional deviations are also present: high dynamic entropy (right angular cortex, middle occipital gyrus), decreased resting state functional connectivity (right angular cortex, right inferior occipital gyrus) [340], and distortion in executive, affective, and default mode networks [341].

## 4. Modelling Approaches

*This section presents some of the most common approaches in brain modelling as a response to the previously discussed pathological effects in the brain, pondering their advantages and drawbacks and introducing some common and novel tools.*

As has been discussed, obtaining an accurate model of the brain’s pathologies is a challenging endeavour due to its inherent complexity. Among other variables, we need to take into account individual variation, evolution over time, and induced damage. Accordingly, any model striving for a precise portrayal of its functioning must be flexible, cohesive, and complete. Connectomics aims to build a structured method to study the brain as a combination of all its internal neural (and glial) networks. Attempting to reproduce the connectome’s structure and function requires a data-rich model that is imperatively multiscalar in space (from synaptic level to the whole brain) and time (from signal processing to complex reasoning and memory).

For the brain, the microscale concerns cell-level phenomena (synapses, membrane polarisation, neurotransmitter releases, etc.), the mesoscale involves neural networks within brain regions and their interactions, and the macroscale implies brain structure and activity as a whole and in synergy with its conditioning environment—yielding so-called whole-brain models (WBM) [342,343]. However, measures obtained by common methods (CT scans, DTI, (f)MRI) are often too broad to cover the meso- and micro-scales. Studies about synaptic processes are (relatively) easily obtainable, but they are globally meaningless when devoid of the network context they partake in.

Of course, the large amount of neurons ($O(10^{11})$) [344] and synapses ($O(10^{15})$) [345] in the human brain makes it computationally unfeasible to transpose or extrapolate individual neuronal function directly onto the macroscale; so, some compromise must be found. Regarding time scales, neuron spiking takes milliseconds. Thus, fast computing is pivotal for any real-time simulation attempts. On top of that, nonlinear transient events occur in biological neural networks [346].

In general terms, there are two traditional ways to tackle multiscale modelling that can be applied to the brain: (1) bottom-up (“direct problem”—from cause to effect, growing in complexity when directly relating local phenomena to global responses); or (2) top-down (“inverse problem”—tracing causes from effects, inverse local inference out of global observations). The latter is the most common, since most measurement techniques (MRI, DTI, CT, etc.) have the whole brain (global scale) as the target, yielding virtual brain models [347]. Such traditional methods, based on the magnetism of the ferrous content in blood’s haemoglobin through the brain’s vascular network [348], do provide snapshots of the brain’s structure and function. There is no doubt that they helped understand the brain better, most importantly in a non-invasive way (no dangerous surgical processes needed). Alas, they do not constitute models by themselves, since they do not allow clinicians to experiment; rather, clinicians can only annotate observed data.

There is a tendency to associate causes with the microscale and effects with the macroscale. Although true in some scenarios, this is not always the case, and assuming so may impoverish models. The modeller must consider macrophenomena with an impact on the microscale as well, such as neurogenesis during brain development. Both direct and inverse methodologies require a great amount of data and a deep understanding of the brain’s functions both ways, which is still in the works. Fortunately, there is a huge amount of available data, although representativity remains an issue. The challenge remains in the limited extent of many of these analyses and the interpretation and generalisation of their results to an archetypal “human brain”. Although network hubs seem to be quite consistent and homogeneous in healthy individuals [349], this is not the case for patients [350,351]. Besides, such generalisations do not provide the whole dynamical picture of functional connectivity [352,353].

Another fact that must be dealt with, as is the case with many other complex systems to be modelled, is surjection: if the model were given by a function y=f(x), there would be many input possibilities $x_{i}$ yielding the same global observation *y*. This phenomenon is also known as neurodegeneracy, a direct consequence of brain redundancy and the nature of graph networks themselves—where many paths could join two different nodes through very different sets of edges [347].

While data in vivo have grown in quality and availability, high-fidelity brain mock-ups could prove quite convenient for a more accurate, non-invasive (in silico) diagnosis and treatment. Prognosis and differential diagnosis remain two complicated questions that still rely mostly on statistical data, which could be biased and/or incomplete, as previously discussed. Models must be sensitive to structural–functional decoupling [311,354] as well, a neurological reality resulting from the brain’s evolutionary adaptability to disturbances (injuries, disorders).

In spite of the advantages of connectomics, the researcher must not reduce a field as complex as neuroscience to a mere topological study; hence, it is a dire necessity to incorporate the biological [355] and chemical considerations behind the modelled phenomena to enrich connectomics through different scales [356]. Furthermore, modelling the brain as an isolated organ is a mistake. Brain function is constantly influenced not only by external stimuli but also by internal factors (e.g., peripheral hormones or metabolic signals), both of them playing crucial roles in pathological states. Thus, a modern connectomics that could change current brain paradigms is necessary.

After this collection of challenges and difficulties (among many others) faced by brain modelling, a short presentation of different proposals follows, each one from their own perspective and thus concerning different types of data, tools and objectives.

### 4.1. Electrophysiological/Haemodynamical

Electrophysiology studies the electrical behaviour of biological tissue in an almost non-invasive manner (no need for surgery in humans). Modelling the connectome as an electric circuit seems self-evident, considering that neurons act as membranes (electrically excitable cells). Electrophysiology is the medical discipline responsible for the analysis of such electric signals, typically through electroencephalography (EEG), and it yielded some of the first attempts at brain modelling, mainly describing synapses [357] and their nonlinear effects [358]. It can provide useful information about many of the mentioned types of brain pathology, e.g., intellectual disability [359].

Electrophysiology provides an advantage over other techniques since electrical excitation can be induced artificially, allowing for measures in vitro and in vivo. Neuromorphic hardware (“neuroinformatics”) is the basis of artificial intelligence—albeit with notable simplifications from the real brain’s circuits. Further explanations can be found in [360,361] and Section 4.3. This is directly related to electrical brain stimulation (EBS), which can alter neural activation thresholds and be measured in diverse ways (evoked potentials) [362]. EBS is used in therapy (e.g., treatment for movement disorders like Parkinson’s [363]) and surgery—tests after tumour extraction, for instance [364]—though parameters must be carefully chosen to avoid further damage [365]. EBS provides direct information regarding functional connectivity across different scales and using varying methodologies [366], although structural connectivity can also be inferred from functional observations or the lack thereof.

Electrophysiological and haemodynamical mappings are intertwined and often complementary. The available imaging techniques rely either on electrical excitation (EEG, MEG, TMS, NIRS) or tracking iron contained in blood’s haemoglobin (haemodynamics: fMRI, CT, PET, SPECT) [84]. Therefore, the electromagnetic theoretic framework links both phenomena (Maxwell’s laws, etc.). Whereas the former showcases an electrical neuronal circuit—membrane activity, i.e., synapses—the latter provides the vascular structure feeding the brain, them being, of course, correlated to each other. Haemodynamic measures are especially useful to detect angiomas (abnormally overgrown blood vessels), which are themselves benign but perhaps surround a malignant tumour.

Task-driven models focus on the activation patterns observed via fMRI, EEG, or a combination of both [367], while subjects perform a given activity under a controlled environment, usually obtained via electrophysiology. They yield partial observations that, superposed on top of one another, form a global—yet not fully integrated—functional connectivity chart. Nevertheless, as with any physical testing, these techniques have some accuracy and representativity issues, stemming both from the design of experiments (small, skewed samples) and the instrumentation-induced uncertainty. Signal analysis tools (e.g., spectral techniques [368] and Fourier’s transform [369,370]) are used to extract useful information out of EEGs. Some authors even argue that the brain could perform a simplified Fourier analysis on its own [371,372].

Several authors note that profound changes must take place in the field to address challenges like performing methodological comparisons and obtaining neurophysiologically meaningful conclusions. This is a call for a unified terminology and biophysical cognitive models that are able to capture multiscalar interactions within the brain [373]. Although novel methodologies like Neuropixels probes [374] allow for an ever-increasing brain region coverage and accuracy, any measurement relying on physical media will always entail some margin of error.

### 4.2. Biomechanical

Despite being protected by the thick, hard skull around it, brain tissue is a very soft material that is 30 times less shear-resistant than silicone gel [375]; i.e., it is compliant [376], with feeble and nonlinear mechanic properties such as loading rate dependency [377,378]. It is also fragile, heterogeneous [379], biphasic (80% water), and scarce in practice (medical availability) [380]. As already mentioned, brain damage does not require skull breakage (that only occurs in penetrating brain injury, which is a small subset of all eventualities). Accelerations, swift turns, external impacts, and diverse conditions (infections, toxicity, cancer, etc.) can wreck havoc within the brain, both structurally and functionally—the latter most frequently being a consequence of the former. All these events may influence the brain’s mechanical properties and neural connectivity (structurally and functionally), yielding valuable information for diagnosis and treatment monitoring.

Biomechanical models try to explain the brain from a mechanical point of view—kinematics, dynamics, etc.—within biologically plausible parameters, since brain tissue is subjected to phenomena unseen in inert materials (namely, physiological functions: birth, growth, death) or affected by them in a different way than inorganic materials (biochemistry). While most of these effects (especially biological processes) outgrow the mechanical perspective in itself, they do have an influence over (bio)mechanical properties; thus, they must be taken into consideration, which is not straight-forward in the slightest. Brain growth, for instance, has been characterised as a morphological process during development [381] and through micro-structural modelling as a soft tissue [382,383], which is not limited to pure kinematics.

On top of biological considerations, the brain possesses some mechanical properties which make it more complicated to model than many bulk materials. For instance, stiffness varies in time and space: it increases globally with age, especially during cortical folding formation (modelled as mechanistic growth [381,384]). Some brain regions are more robust than others [385], being positively correlated with collagen [386] and myelin content [387], with the latter being also used as a marker for disease [388]. Deformations are usually not homogeneous either [389], except in unconfined compression tests, whose validation require an estimation of the friction coefficient, which is again variable with the loading rate [390]. Besides, measurements themselves are quite complicated to perform: they are costly and conditioned by conservation [391], preparation [392], and the testing environment [393]. Uniaxial testing is quite difficult due to extreme softness; hence, shear testing is more feasible and more common [375,377]. Thus, conceiving brain tissue as a homogeneous, linear, and/or isotropic material, although convenient for simplicity, could never produce any high-fidelity model.

Interestingly, despite all this variability, two fundamental biomechanical principles hold true when modelling soft biological tissue: tensional homeostasis and turnover [394]. The first concept can be derived from the explained Monro–Kellie principle [119]. It states that soft tissues (brain, arteries, etc.) have a preferred “homeostatic” loading state and trigger biological responses (segregation of certain biomolecules by the cell) to keep the state consistent. This explains why, across several ages and species, intracranial pressure remains constant at around 2–4 N/m per lamellar unit [394], yielding a Cauchy stress of 150 to 300 kPa, only altered as a result of an unforeseen (TBI) or planned physical intervention (surgery) [120,376] or disease [395]. The second pillar does not apply to inert bulk materials, just to living organisms. Cells forming soft tissues, including the brain, grow, reproduce, interact with their environment (neural migration [78,79,80]), and die, which creates a mass variation known as “turnover”. This phenomenon, as with many others in the brain, is multiscalar in time (minutes to months) and space (cellular or extracellular). Turnover can be balanced (homeostatic, healthy individual) or skewed (adaptative or pathologic), either positively (fibrosis) or negatively (atrophy) [394].

The attempts to explain growth and remodelling in biological soft tissues in terms of mass–stress interactions yielded the so-called constrained mixture models [396,397]. From a purely mechanical perspective, brain tissue exhibits nonlinear mechanical responses—its stress–strain curve is approximately exponential. Additionally, considering the aforementioned strain rate dependency (and thus, time dependency), viscoelastic models [398,399] or a combination [400] are preferred over solely hyperelastic ones [117,401]. These kinds of models can be useful when evaluating the consequences of physical brain trauma—brain injury, that is—and setting the mechanical limits to avoid, as well as providing a model for damage evaluation and treatment follow-up. However, different methodologies, datasets (sample size, patient age [402], species, and environmental factors (namely, humidity [403] and temperature [391,393])), and theoretical frameworks yield varying mechanical results, even spreading across orders of magnitude.

Full mechanical studies of the human brain [378,404,405] are complex and costly, and they frequently fail to consider the influence of the angiome [386] (dependence on haemodynamical models) in mechanical (e.g., stiffness induced by anoxic necrosis) and organisational aspects (network connectivity). Isochoric and (quasi-)incompressible mechanical models (e.g., Hencky’s) pose a plausible approximation [406]. Such approaches provide clues for diagnosis and/or treatment that cannot be easily obtained via medical imaging, e.g., diffuse axonal injury [117,379]. Biophysical models, especially those allowing for brain activity tracking, can greatly enhance diagnoses of neurodegenerative illnesses as well [226,407]. The mechanical testing of brain tissue remains a complicated feat by itself and is further hindered by the scarcity of brain tissue, although non-invasive techniques such as ultrasounds—namely, shear waves [408,409]—pose an interesting option to perform in vivo mechanical measurements.

### 4.3. Mathematical

These models try to depict the brain through mathematical tools, i.e., calculus, algebra, statistics, information theory (IT), etc. This task proves challenging considering its already explained multiscalarity (space- and time-wise), complex biochemical processes, and the difficulty that its testing entails. Thus, obtaining purely analytical expressions for such complex biochemical phenomena is extremely challenging—if possible at all. Mathematical models attempting to convey this complexity often lack these considerations, or limit themselves to mere statistics, which is very common in medicine in general and neuroscience in particular. Unfortunately, statistics yields very different (and even contradicting) results depending on the used sample(s), which can contain an unwanted bias induced by its size, variance, and/or data collection techniques.

Important assumptions about the model’s work variables are made to tackle this inherent brain variability, the most bold being homoscedasticity, i.e., the homogeneity of variance, and normality, i.e., following the normal distribution. Along with nonlinearity and outlier interpretation, these are the foremost problems in traditional statistical approaches, ending in false positives (Type I errors) and broader accuracy concerns [410]. If the modeller wishes to produce robust models, any statistical method exclusively based on mean and/or variances must be ruled out by default. More practical, data-driven interpretations—thus, free of theoretical assumptions and problematic generalisations—have emerged to solve these problems, such as statistical learning, which is focused on data-inferred interpretations, i.e., building theory on a purely empirical basis [411].

In the past, the brain’s adaptive and beneficial variability in neuronal morphology [412,413,414,415] has perhaps been addressed with limited statistical tools and frequently from a static perspective [416]. Stochastic/heuristic approaches are available for this purpose, such as Boolean networks [417]—wherein Boolean logic junctions act as stochastic surrogates for brain activation dynamics—and, most commonly, agent-based models [418,419]. The latter provide a more flexible framework, where an agent (e.g., a migrating neuron) interacts with its environment, reaching equilibrium points (homeostasis) within complex dynamic settings. Since it allows for quick decision making [420], this technique has been used to simulate different biological aspects: neural community interactions [361,421,422] and pathological contagion [423] and evolution (tumour-induced angiogenesis [424], glioblastoma multiforme [425]).

Importantly, model variables, whether qualitative or quantitative, are abstract concepts—often expressed through mathematical language, but ultimately chosen by the modeller, and, thus, subject to their bias. This is true for many types of models, but especially mathematical ones. Some proposals provide parameter-free statistical testing to alleviate such subjectivity [426]. Neuroscience often deals with many user-defined input and output variables, with many of them not even considered in the first place or difficult to measure. This greatly hinders analytical models, so some order reduction techniques may be needed to avoid the so-called “curse of dimensionality”: too few variables may yield an easy-to-implement but too simplistic model, whereas too many produce complex, costly models that may not even offer accurate results. There are two groups of tools to tackle this inconvenience: model order reduction and machine learning.

First, model order reduction (MOR) [427] is the procedure by which a model gets “compressed” into a lower, more manageable resolution that still conserves its more defining features, conveying its underlying behaviour. These techniques are mainly algebraic, redefining the full system into a reduced version represented by their greatest eigenvalues (principal component analysis) [428] or user-defined modes (proper orthogonal/generalised decomposition) [429,430,431]—which involves taking lower resolution snapshots representing the evolution of the system—or locally overcoming nonlinearities (locally linear embedding) [432]. A more detailed overview on MOR can be found in [433].

Second, machine learning (ML) is the process allowing a programmed machine (i.e., a computer) to obtain results (outputs) when given certain data observations (inputs). Neural networks in particular have proven to be a way to circumvent the issue for now, since they capture the brain’s typical nonlinearities without the need for an analytical expression. ML models can forecast nonlinear outcomes even if the system—i.e., its fundamental variables—are not known [434]. Emulating neural efficiency in diverse tasks, namely, learning, has been a question since the dawn of information theory, crystallising in the McCulloch–Pitts neuron model. Their “perceptron” [435], organised in layers (input variables, hidden dense—i.e., fully connected—layers, and output targets), aims at a stochastic, biologically inspired regression, like that of least squares. This allows for the reproduction of some brain features like metastability and other related phenomena (e.g., Sherrington–Adrian observations [436]). The Dartmouth Workshop in 1956 [437], ignited by the perceptron and Turing’s notions on ML [438], can be cited as the official birth of artificial intelligence as such.

Although very useful for multiple applications, experts soon pointed out important discrepancies between actual biological neural networks and their intended artificial mock-ups—namely regarding backpropagation—in the way that errors are corrected from the outputs to the inputs to yield an accurate prediction [360], which constitutes the basis of regression in ML. There are several fundamental differences between artificial (ANNs) and biological neural networks (BNNs), such as the backpropagation procedure (layered vs. nonlayered), neural plasticity (functional vs. structural and functional), and metastability (occasional byproduct vs. biological requirement); see Section 2.5 in [361] for a more detailed explanation. A simple multilayer perceptron (MLP)—a generalisation of McCulloch–Pitt’s neuron with multiple intermediate layers—will not be enough to mimic the brain.

The biggest issue that biological models face—especially in the brain—is, simplifications and generalisations aside, computing power. As previously said, the number of neurons in a typical adult brain is in the hundreds of billions, while synaptic processes ascend to the thousands of trillions. These scales are unmanageable even by the best-performing computers available today, considering the best existing microprocessors contain around $10^{12}$ transistors and hundreds of km of wire, whereas the human brain processes around $10^{14}$ synapses through 3$\cdot 10^{5}$ km of neural pathways (longer than the Earth–Moon distance) [439]. The advent of quantum computing may provide a feasible solution, given its similarities with the biological brain’s functioning [440,441]. Neuromorphic computing, also known as neuroinformatics, takes inspiration from the human brain’s neural architecture to produce hardware and software [442] inspired by the brain’s efficiency [443], using qu-bits which aim to replicate superposing signals (metastability) [444].

In the meantime, ML techniques are already being used to model brain activity. Convolutional neural networks (CNNs) tackle datasets upon which convolution (the averaging of neighbouring values through the scalar product of a kernel and the inputs) can be performed, i.e., matrices, proving especially effective when dealing with recognition, detection, and/or segmentation tasks. Most importantly, this allows for the analysis of medical imaging such as MRI [445,446], e.g., finding unnoticed patterns which could help identifying [447,448] and classifying [449] prodromal brain tumours or Alzheimer’s [450].

Spiking neural networks (SNNs) [451,452,453] seem to capture the brain’s behaviour in a more biologically plausible manner, aiming at real-time functional connectivity [454]. Their performance improves if they are enhanced by synaptic plasticity functions, namely spike-timing-dependent (STDP) [455,456,457] instead of the regular gradient descent. They allow for the inclusion of structural plasticity as well [455]. SNNs can be combined with quantum neuromorphic architectures [458,459] to enhance their versatility and information processing power.

Graph (neural) networks are also pertinent because of their ability to capture the network’s topology—that is, structural connectivity [460]—and include it into the information flow, with the backbone of graph theory behind it [238,312,461,462,463]. Some examples have already been cited, providing promising results [28,31,32]. GNNs can be further empowered by considering the fractal nature of dendrites, again a result of evolutionary structural and functional adaptability [464].

There are many other machine learning and manifold learning techniques, which interested readers can find in [465].

### 4.4. Application to a Model of the Boolean Logic Behaviour of Neuronal Self-Organised Communities

**Figure 2:**
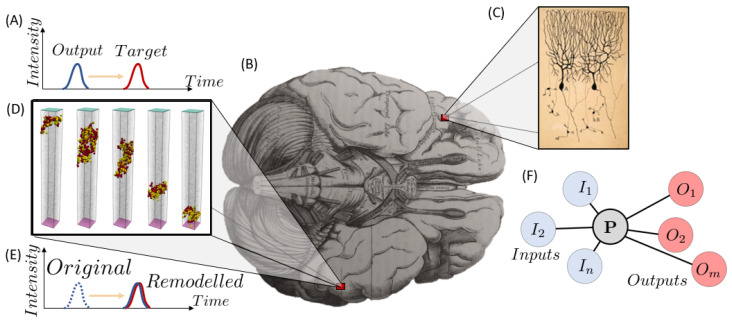
The preliminary results show that it is feasible to create an agent-based model to reproduce the communitarian behaviour of neurons, which are capable of modulating a signal (**A**,**D**,**E**) through plastic remodelling and the inhibition of the cells. The neurons propagate the signal from the stimulation at the blue square (**D**) to the signal received in the pink square at the bottom. In (**D**), different propagation stages are shown. The digital twin of a neuron is shown in (**F**), with the inputs and outputs. All illustrations are made with a drawing of the brain (**B**) found in Gray´s Anatomy [466] and a drawing of the neurons (**C**) by Ramón y Cajal.

This subsection is intended to point out the modelling implications of the diseases summarised in this review in a cognitive agent-based model [361]. This model is an extension of an ABM model [424,425] of brain cells where the modelled neurons can interact, migrate, connect, and perform plastic remodelling. The details of the model are in Irastorza-Valera et al. [361] (see Figure 2 for a visual diagram of the presented model), and it is a novel treatment of the McCulloch–Pitts neuron as a living cell capable of interacting with its surrounding community. That implies that the neuron may be affected by environmental mechanical abnormalities (brain damage) through a disconnection or reduction in neuronal connections. As well, the neuronal connections may be weakened or diminished through neurological disorders, and the connections and tendency to associate with each other may explain the psychiatric disorders.

This is a framework to implement and study different diseases in silico, as well as potential treatments.

## 5. Conclusions and Future Research Lines

*This closing section mainly contains some concluding remarks, a short summary of the article’s content and some suggestions for research opportunities to be explored in subsequent works.*

This review article highlighted the need for accurate brain modelling for monitoring pathologies and described common challenges in brain mapping and how they might be faced. First, the motivations for such a need are explained: brain-associated illnesses take a huge social and economical toll globally—which is projected to grow in the following decades—and the current tools to face them have proven insufficient. A short overview on the state of the art in brain mapping follows, explaining how current tools are limited and how new emerging theoretical (connectomics) and methodological frameworks (e.g., data science) can help answer the field’s many open questions. The main section consists of a carefully written list of brain pathologies accompanied by succinct medical descriptions, focusing on identifiable causes and consequences related to brain connectivity. After that, several existing modelling tools and approaches through different lenses (medical, engineering, mathematics) are presented, with consideration of how they can contribute to ease the aforementioned issues.

As explained throughout this article, brain modelling requires very diverse insights from varied areas of knowledge, ranging from the most obvious ones (neuroscience, chemistry, biology) to some others whose implications are less evident (mathematics, physics, information theory, mechanics). Such an interdisciplinary approach is rather challenging and demands collaboration between experts in all involved fields of knowledge, which are many and far apart. Nevertheless, each attempt—humble as it may be—strives a bit closer towards a global solution. Fruitful synergies can be fostered between physicians (neurosurgeons and neuroscientists, yielding haemodynamical observations), engineers (biomechanical models), and mathematicians and data scientists (mathematical tools).

All these scientists must push to extend such models to include all central and peripheral nervous systems, considering the role of glia, myelin, the angiome, and other biochemical actors. As for the brain itself, special attention must be paid to the cortex (most synapses within 20% of the brain’s volume) and cerebellum (80% of all neurons contained in 10% volume), whilst not forgetting interconnected subcortical regions [67]. The interaction with the environment (external stimuli, community) and internal factors (peripheral organs) must be taken into account as well. Nowadays, the development of new technologies used in neuroscience, both in animal and human models, allow us to track specific brain functions from genetics to behaviour, thus multiplying modelling possibilities but also making it more challenging. From the neuroscience perspective, it is essential to consider all variables considered above in order to obtain a clear model of the pathophysiology of the brain.

An ideal objective would be the customised modelling and diagnosis of each neurological patient (individual, real-time brain mapping). An emphasis on preventive monitoring and more accurate diagnoses is ubiquitous in engineering in general, materialised by so-called digital/hybrid twins [467]: totally data-driven and/or physics-enhanced mock-ups of dynamical systems able to provide real-time and accurate responses, including self-corrections [468]. This is an ambitious and complex objective, which may take time to fully develop. For the time being, the improvement of existing methodologies through these modern tools and the completeness of the theoretical neuroscientific framework—perhaps with these very tools—constitutes a reachable and valued mission for patients.
